# Predictors of child resilience in a community-based cohort facing flood as natural disaster

**DOI:** 10.1186/s12888-020-02944-y

**Published:** 2020-11-19

**Authors:** Muhammad Arshad, Muhammad Kashif Mughal, Rebecca Giallo, Dawn Kingston

**Affiliations:** 1 Faculty of Nursing, University of Calgary, Calgary, Alberta Canada; 2 Center for Genomics and Systems Biology, New York University, Abu Dhabi, United Arab Emirates; 3 Murdoch Childrens Research Institute, The Royal Children’s Hospital, Parkville, Victoria Australia

**Keywords:** Child resilience, Flood, Natural disaster, Mental health, Child development

## Abstract

**Background:**

Natural disasters are unpredictable and uncontrollable events that usually induce significant level of stress and social disruption in afflicted individuals. The consequences are formidable, affecting lifetime health and economic prosperity. Among natural disasters, floods are the most common causes and tend to have the highest economic burden. The aim of this study was to examine factors associated with child resilience in the face of the natural disaster experienced by the city of Calgary, Alberta, Canada during its unprecedented flood of 2013.

**Methods:**

The current study was conducted in a community-based cohort situated in the city of Calgary. The participants were recruited out of the All Our Families longitudinal cohort within the Cummings School of Medicine at the University of Calgary. Of the total 1711 people contacted, 469 people consented and completed questionnaire. Of those 469 who consented to be part of the study, 467 were eligible to be included for analysis. A flood impact questionnaire was delivered 6 months after the 2013 flood in families whose children were an average of 3 years old. Mother reported questionnaires were used to assess child resilience. The study included maternal data on a range of factors including socio-demographic, history of mental health, relationship with the partner and social support. Child related data were also incorporated into the study, and variables included delivery mode, child sex, and child age at the time of disaster.

**Results:**

Child resilience was best predicted by mother’s age and social support, and by child gender, the child’s externalizing and internalizing behaviors and the Rothbart temperament scale: effortful control. Furthermore, this study revealed that children who were more exposed to the flood events, showed higher resilience compared to the children who were less or not exposed.

**Conclusions:**

These findings highlight the risk and protective factors that predict child resilience and suggest that mother reported questionnaire are useful tools to assess child resilience amidst early life adversity.

## Background

Children exposed to natural disasters are at risk of depression, anxiety, post-traumatic stress disorder (PTSD) behavioural disorders, and developmental/learning problems [1–3]. Floods are among the most common natural disasters that cause physical and economic impacts which may trigger a wide range of mental health problems such as PTSD, depression, and anxiety in adults [4] and children [5–7]. Reports of the impact of flooding events such as 2011 Brisbane floods in Australia, and 2007 floods in United Kingdom showed that flood-impacted respondents had higher levels of mental and psychological problems than those not impacted [8, 9]. Similarly, parental distress is one of the common risk factors and is linked to negative child psychopathology in the aftermath of a natural disaster. Other contributing factors that influence outcomes of adversity include the home environment and socioeconomic status [10, 11]. The negative effects of such disasters vary in the families and children. Mental health of some children is negatively impacted more than others amidst natural adversities, and what differentiates them is not well understood.

A significantly increased level of mental health symptoms was reported among the children that were exposed to Hurricane Katrina for example; more than 60% of children showed elevated PTSD symptoms [12]. Results from the 2010 Chilean Earthquake indicated that children that experienced the natural disaster scored poorly on some early language and pre-literacy assessments than those who did not [13]. Over the past decade, there have been an upsurge in studying resilience in individuals, families and children [14–16]. In the current global scenario, there is an urgency to research various new adversities to better understand resilience [14, 17, 18].

### Definition of resilience

American Psychological Association defines resilience as “the process of adapting well in the face of adversity, trauma, tragedy, threats or even significant sources of stress” [19]. Resilience is often conceptualized as an outcome involving two parts: 1) experiencing adversity operationalized in many ways, such as wars, floods.; and 2) achieving a positive outcome despite the specified risks [20, 21]. A number of individual (e.g., temperament, adaptive skills), family (parenting relations, support outside the family), and environmental (fairness of opportunity, social justice) factors promote resilience [22]. While these factors are important, resilience is contextual, may change over time, and is often considered as a phenomenon- not a trait. For example, an individual may show resilient outcomes in one context, but not in other [23].

Studies on resilience suggest that level of exposure to an adverse event can influence short and long term physical and mental health outcomes. With major advances in technology, these outcomes can be investigated at multiple levels of analysis including epigenetic changes, stress regulation, and relationship between parent(s) and children [24]. Clinical researchers have identified several salient protective and risk factors of resilience and mental health. For example, major risk factors identified in families and children following natural disasters were displacement, separation from caregiver, lack of social support, environmental concerns, economic difficulties, disruptions to infrastructure, and history of mental health [4, 25–27]. Protective factors mitigate the potential adverse mental health consequences of a disaster on individuals, children and families [26]. Several protective factors associated with child resilience include caring family, close relationships, skilled parenting, self-efficacy, positive view of the self, connections with well-functioning communities and social support [24]. A recent systemic review by Gartland et al. [28], described an association of a range of child, family, school and community factors with resilient outcomes. Furthermore, authors linked resilience with improved cognitive skills, emotion regulation, relationships with caregivers and academic engagement in children [28]. Although there are studies available on negative impacts of different adversities on child mental health, [5, 12, 13, 29], to our knowledge, this is the first study on child resilience amidst flood in the context of a community based sample.

### The present study

An opportunity emerged to conduct community-based cohort study for natural disaster, when Calgary experienced its worst flooding in 2013. The focus of this study was to examine factors associated with emotional resilience among children who were exposed to the flood. The participants were recruited out of the longitudinal All our Families (AOF) cohort [30] where we had an opportunity to draw upon data from this cohort from pregnancy to 3 years postpartum. At the time of flooding, the average age of children was 3 years, and ranged from 1 to 4 years. The flood impact survey was administered 6 months after the flood and 1711 participants completed the survey. The families who consented to participate in the survey were subsequently sent an electronic survey to assess child resilience [31].

The study included maternal data on a range of factors including socio-demographic, history of mental health, relationship with the partner and social support, and these factors are considered to have an impact on child’s health later in life [25, 26]. Child related data were also incorporated into the study, and variables included delivery mode, child sex, child age at the time of disaster, children displaying internalizing and externalizing behaviour problems [32, 33]. The objectives of the study were to identify predictors related to child resilience in a community-based sample facing daily life stressors and a natural disaster (Calgary Flood 2013) while accounting for the preceding pre-disaster risk factors such as mother’s history of mental health, low family income, and mother’s education.

## Methods

### Study design and recruitment

The data for this analysis was taken from the All our Families (AOF) cohort- formerly known as All Our Babies (AOB) study- and the Prediction and Understanding of Resilience in Albertan Families: Longitudinal Study of Disaster Responses (PURLS). The AOF is an ongoing, longitudinal pregnancy cohort of women and children that began in 2008 in Calgary, Canada. The main objective of AOF cohort is to study the life course investigation of the relationships between early life adversities (e.g., adverse maternal health, preterm birth) and child development. A detailed description of the AOF cohort, study design, and samples are described in the previously published study [34]. The questionnaires used in the current study were published elsewhere [31, 34]. Briefly, participants were recruited from primary health care offices, the Calgary Laboratory Service, and community posters. The participants completed three questionnaires, at study intake (< 25 weeks gestation), between 34 and 36 weeks, and 4 months postpartum. Participants who consented for future research completed follow-up questionnaires when their child turned one, two, and 3 years old. The AOF participants affected by Alberta floods in 2013 also completed the flood surveys questionnaire. The eligibility criteria for the AOF study were aged 18 or older, less than 25 weeks pregnant, receiving prenatal care in Calgary, and proficient in reading and writing English [33]. Majority of the respondents were born in Canada, married and belonged to Caucasian ethnicity, had higher income (>$CAD 60,000 per annum) and education. Moreover, the participants were either pregnant women and/or mothers of young children [31, 34].

The PURLS resilience study was a sub-study of AOF cohort that was started in September 2016. The main goal for the PURLS resilience study was to study the relationship between maternal factors with child resilience in the face of common life adversities and Calgary flood using a combination of psychosocial, biological and genetic data, and detailed protocol for this study has been published [31]. Briefly, the PURLS resilience survey was emailed to the eligible PURLS participants between September 2016 and October 2017. Of the total 1711 people contacted, 469 people consented and completed questionnaire, giving an overall recruitment rate of 28%. All those who completed the PURLS resilience questionnaire were considered for the analysis. In those 469 who consented to be part of the study and completed at least some of the questionnaire, 467 were eligible to be included for analysis [31]. Majority (67%) of the children at the time of flood questionnaire (Table 2) were 36 months old or more. This questionnaire was filled by the participants in 2013 at the time of Alberta Flood. However, the DESSA measure was a part of the PURLS study and was completed after September 2016 when most of the children in the AOF cohort turned 5 years or more. Mother reported questionnaires were used to assess child resilience. This study was approved by the University of Calgary Conjoint Health Research Ethics Board (#REB16–0133).

### Measures

For this study, data were selected from three categories of the AOF study: 1) maternal/family variables (socio-demographics relationship status, history of mental health, social support, and partner relationship during pregnancy); 2) child variables (mode of delivery, child sex, age at disaster, Rothbart temperament scale: effortful control, internalizing and externalizing behavior problem at 3 years postpartum); 3) flood co-variates.

Child Temperament was assessed using the 36-items very short form (CBQ-VSF) [35]. The CBQ-VSF is a caregiver reported measure intended to provide a comprehensive assessment of temperament in children aged three to 7 years. Responses for all items are scored on a 7-point Likert scale ranging from 0 (extremely untrue) to 7 (extremely true). The internal consistency for the CBQ- VSF in our sample ranged from 0.21–0.68 and for NLSCY ranged from 0.51–0.75. CBQ assesses child temperament on three broad temperament traits: Surgency/Extraversion (SE), involving the propensity to act with impulsive and active behavior which includes positive affect; Negative Affect (NA), referring to the predisposition to experience negative feelings and difficulty being soothed; and Effortful Control (EC), which involves self-regulation, including voluntary regulation of attention and behavior [36]. The internal consistency for these constructs ranged from 0.62 to 0.78 [35]. Children’s behavioral functioning (externalizing and internalizing) was assessed using 25-items Behavior Scales developed for the Canadian National Longitudinal Survey of Children and Youth (NLSCY) [37]. Items for the NLSCY Behavior Scales were built on items from the Child Behavior Checklist (CBCL) [38]. The NLSCY Behavior Scales is a caregiver reported measure and items are scored on a 3-point Likert scale of not true to very true or often true. The NLSCY scale comprise four subscales, including two externalizing subscales (hyperactivity and inattention; physical aggression) and two internalizing subscales (emotional disorder and anxiety; separation anxiety). The internal consistency for the subscales ranged from 0.58 to 0.80 [33].

In this study, flood impact was measured using one flood question that was scored on a 5-point scale with an increasing order, for example, question reads; “Overall, on a scale of 1-5, how much impacted were you by the flood? For each item, 1 = not impacted at all, and 5 = very much impacted. Flood impact question was recoded into three categories. For example, 1 & 2 were recoded as “not impacted”, 3 & 4 as “somewhat impacted”, and 5 was recoded as “very much impacted”.

### Child resilience

The Devereux Student Strengths Assessment (DESSA) is considered as the psychometrically robust and practical scale of social-emotional functioning in school and out-of-school-time settings. In this study, child resilience was measured using the DESSA, which is a 72-item scale designed to be used on children from ages 5 through 13 years (kindergarten to grade 8) to measure their social-emotional competencies using a strength-based approach. Internal consistency coefficients estimated for the DESSA Social-Emotional composite and eight subscales ranged from 0.82 to 0.99. Test-retest reliabilities ranged from 0.79 to 0.90 (parents), and 0.86 to 0.94 (teachers/staff) [39]. Most recently, several studies have used the DESSA as a proxy to measure resilience in children with various mental health problems [40, 41].

Each question is scored on a 5-point Likert scale, for example, question reads; “During the past four weeks, how often did the child....” and then rates each item from 0 to 4 (0 = never, 1 = rarely, 2 = occasionally, 3 = frequently, 4 = very frequently) that can be summed then re-weighted into 8 subscales: self-awareness, social-awareness, self-management, goal-directed behaviour, relationship skills, personal responsibility, decision making, and optimistic thinking. The re-weighted T-scores for each subscale can be summed and re-weighted into a new T-score for a total social-emotional scale. A higher T-score indicates greater social-emotional competence. The internal consistency for DESSA subscales in our sample ranged from 0.83–0.91. The internal consistency for the DESSA subscales in the sample was recorded as follows: self-awareness (0.84), social-awareness (0.87), self-management (0.88), goal-directed behavior (0.87), relationship skills (0.91), personal responsibility (0.86), decision making (0.88) and optimistic thinking (0.83).

### Predictors

The study predictors were categorized into four groups: (1) demographics, (2) history of maternal mental health and partner relationship variables during pregnancy (3) child variables and (4) flood disaster covariates. Group 1 and 2 variables; demographics and history of maternal were taken from AOF time points 1 (under 25 week’s gestation). Group 3, child variables were taken at 4 months postpartum, and 3 years postpartum. Group 4 variables were taken at 3 years postpartum.

Group 1 variables are: relationship status as measured during pregnancy (married/ common law/living with a partner vs. single/separated/divorced/widowed), maternal age at delivery (< 25 years vs. > 25 years), education measured as during pregnancy (high school or less vs. some post-secondary or more), ethnicity (non-white vs. white), family income per annum as measured during pregnancy (CAD$) (< $60,000 vs. ≥ $60,000 per year).

Group 2 variables are: maternal perceptions of adequacy of social support assessed using the MOS-SS (score of < 70 vs. ≥ 70), history of mental health and partner relationship (has partner but there is at least some tension in the relationship vs. has a partner and there is no tension in the relationship).

Group 3 variables are: delivery mode (caesarian vs. vaginal), child displaying internalizing and externalizing behaviour assessed using the NLSCY Child Behaviour checklist (high risk (1SD above the mean or higher) on either the hyperactivity inattention subscale or the physical aggression subscale vs. low risk (less than 1SD above the mean) on both the hyperactivity inattention subscale and the physical aggression subscale), child’s effortful control, measured using the Rothbart Child Temperament Scale (RCTS) effortful control subscale (low effortful control, scored mean - 1 SD or less vs. medium/high effortful control, scored greater than mean – 1 SD), child’s age at the time of completing the flood questionnaire (24–36 month vs > 36 months), and Child’s sex (male vs. female).

For group 4 natural disaster covariates: the disaster covariates from the Calgary flood. They were taken from the flood survey and the resilience timepoint (2–5 years postpartum and 4–10 years postpartum). The variables are: 1): impact from the flood; on a scale of 1–5 where 1 = “not at all impacted” and 5 = “very much impacted”, were recoded into a binary variable; at least somewhat impacted (3–5) vs. not at all or very little impacted (1–2). 2): Preparedness for the flood (at least somewhat unprepared vs. well prepared), household was evacuated because of the flood (yes vs. no), member of the household experienced loss of income as a result of the flood (yes vs. no), experienced loss of damage as a result of the flood (yes vs. no), experienced significant threat or danger as a result of the flood (yes vs. no).

### Data analyses

Of all the participants considered for analysis (*N* = 469), 467 completed the DESSA. The overall social-emotional re-weighted scale from the DESSA was used in the analysis as the outcome (continuous variable) to determine a child’s resilience amidst of natural adversity. Analyses were conducted using SPSS v25 software in three stages. First a large pool of predictor variables and disaster covariates were considered for bivariate analysis with the child resilience outcome variable- DESSA overall socio-emotional T score. Variables were selected based on clinical relevance, reporting validity (variables with high percentages of missing data were excluded), and enough variability or category representation (variables with categories that were severely under populated were considered for exclusion). Second, bivariate analysis was conducted between each of the predictors and the resilience outcome variable (continuous), and between each of the disaster covariates and the resilience outcome variable. For the categorical predictors and covariates bivariate analysis was conducted using linear regression models.

Finally, variables significantly associated with the child’s resilience outcome at *p* <  0.05 were entered to a final multivariable model (except mother’s age). All the variables were tested for multicollinearity (Pearson correlation < 0.6) before entering the final model. Results are reported with 95% confidence intervals.

The extent of missing data across all the variables of interest included in the analysis averaged 4.9%. Missing data was handled by multiple imputations in SPSS by imputing twenty datasets under a multivariate normal model. Pooled estimates for all proportions and model parameters were generated and reported in the results. The multivariable regression model was conducted using (a) the total sample with the imputed data and (b) only cases with the complete data. Given that there were minimal differences between the two results, only those using the imputed data are presented.

## Results

### Sample characteristics

A total of 467 women met all eligibility criteria and completed at least part of the child resilience questionnaire. Socio-demographic characteristics of the participants included in the study that were similar to the overall sample of the All Our Babies cohort [33]. Table 1 show that majority of the mothers were 25 years or older, partnered, completed at least some post-secondary education, yearly family income of $60,000 (CAD$) or more, and born in Canada.

**Table 1:** Participant characteristics at ≤24 weeks gestation (*N* = 467 except where data is missing)

Characteristics	n (%)
**Maternal age at delivery**	
25 years or less	46 (9.8)
More than 25	411 (88.0)
Not reported	10 (2.2)
**Relationship status**	
Single, divorced, widowed, other	22 (4.7)
Married, common law, living with a partner	442 (94.7)
Not reported	3 (0.6)
**Maternal education**	
Completed high school or less	36 (7.8)
Some post-secondary or more	428 (91.6)
Not reported	3 (0.6)
**Family income (CAD$)**	
$59,999 or less	63 (13.4)
$60,000 or more	386 (82.3)
Not reported	20 (4.3)
**Country of birth**	
Outside Canada	80 (17.1)
Canada	385 (82.5)
Not reported	2 (0.4)
**Ethnicity**	
Non-white	81 (17.3)
White	384 (82.3)
Not reported	2 (0.4)

### Flood impact and child resilience outcomes

**Fig. 1:**
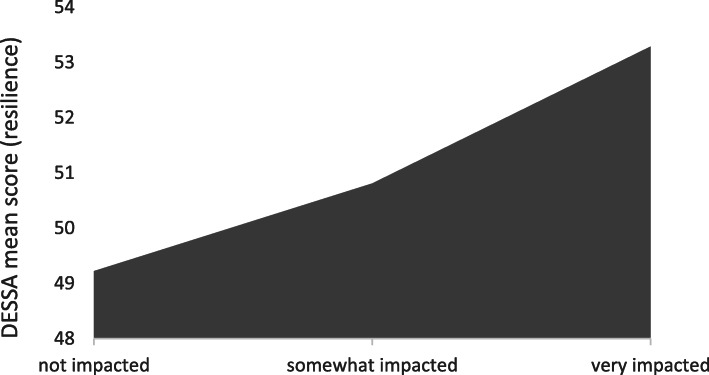
Relationship between flood impact and child resilience. DESSA score was not significantly different among three groups (ANOVA, *p* = 0.20). Mean DESSA score results indicated that resilience increased with the increase of flood impact

Descriptive for the predictors are provided in Table 2. Flood impact scale that ranged 1–5 was recoded into three categories; 1 & 2 were recoded as “not impacted”, 3 & 4 as “somewhat impacted”, and 5 was recoded as “very much impacted”. There were total 355 (76%) children that were not impacted, 104 (22.3%) somewhat impacted and 8 (1.7%) were very much impacted. The DESSA mean (SD) = 49.61 (9.09), scores ranged 28–72 on a continuous scale, and higher score represents more resilience. DESSA score (resilience) was compared among children in the above-mentioned three categories; not impacted, somewhat impacted, and very much impacted. Although not statistically significant (ANOVA, *p* = 0.20), an increase in mean DESSA score (resilience) was recorded in children that were more impacted by the flood (Fig. 1).

**Table 2:** Descriptive for predictors of child resilience and flood disaster covariates (*N =* 467 except where data is missing)

Demographics and maternal, child and flood variables	n (%)
**Partner relationship at 3 years postpartum**	
Some or lot of tension	184 (39.4)
No tension	234 (50.1)
Not reported	49 (10.5)
**Social support at 3 years postpartum**	
Low	90 (19.3)
Adequate	347 (74.3)
Not reported	30 (6.4)
**Previous history of mental health**	
Yes	171 (36.6)
No	294 (62.9)
Not reported	2 (0.5)
**Child’s gender**	
Boy	238 (51.0)
Girl	195 (41.7)
Not reported	34 (7.3)
**Child age at the time of flood questionnaire**	
24–35 months	94 (20.1)
36 months or more	313 (67.1)
Not reported	60 (12.8)
**Delivery mode**	
Caesarian	113 (24.2)
Vaginal	349 (74.7)
Not reported	5 (1.1)
**Child displaying internalizing behavior at 3 years postpartum**	
Yes	98 (21.0)
No	340 (72.8)
Not reported	29 (6.2)
**Child displaying externalizing behavior at 3 years postpartum**	
Yes	103 (22.1)
No	333 (71.3)
Not reported	31 (6.6)
**Impacted by the flood?**	
Not impacted	355 (76.0)
Somewhat impacted	104 (22.3)
Very much impacted	8 (1.7)
**Flood preparedness**	
Unprepared	274 (58.7)
Well prepared	136 (29.1)
Not reported	57 (12.2)
**Did you evacuate household?**	
Yes	23 (4.9)
No	387 (82.9)
Not reported	57 (12.2)
**Did you or someone in household experience loss of income?**	
Yes	57 (12.2)
No	352 (75.4)
Not reported	58 (12.4)
**Experienced threat or danger during flood?**	
Yes	23 (4.9)
No	385 (82.5)
Not reported	59 (12.6)

### Bivariate analysis

The results of the bivariate analysis are shown in Table 3 Model A. Bivariate results suggested a significant and positive relationship of child resilience with mother’s education (post-secondary or above), no tension in partner’s relationship, adequate social support and no history of mental health. Child variables included child’s sex as a girl, child not displaying internalizing and externalizing behaviour, and medium/high effortful control.

**Table 3:** Bi and multivariate analysis for predictors of child resilience (*N* = 467, except where data is missing)

Predictors	Model A (unadjusted)				Model B (adjusted)			
	B	SE	t	*p* value	B	SE	t	*p* value
***Maternal/family variables***								
**Mother’s education**								
Post-secondary	3.67	1.62	2.27	0.02	–	–	–	–
**Maternal age at delivery**								
> 25 years	1.45	1.42	1.02	0.31	2.86	1.42	2.02	**0.04**
**Family income**								
$60,000 or more	1.62	1.23	1.32	0.19	–	–	–	–
**Relationship status**								
Married/common law	0.81	2.03	0.40	0.69	–	–	–	–
**History of mental health**								
No	2.07	0.88	2.36	0.02	–	–	–	–
**Partner relationship during pregnancy**								
No tension	2.30	0.96	2.39	0.02	–	–	–	–
**Social support during pregnancy**								
Adequate	3.69	1.21	3.04	0.002	2.50	1.22	2.04	**0.04**
***Child variables***								
**Child’s gender**								
Girl	1.83	0.89	2.06	0.04	1.57	0.84	1.86	0.06
**Child age at the time of flood questionnaire**								
24–35 months	0.64	1.11	0.58	0.56	–	–	–	–
**Delivery mode**								
Vaginal	1.26	0.99	1.27	0.20	–	–	–	–
Caesarian								
**Child displaying externalizing behavior at 3 years postpartum**								
No	4.09	1.02	4.01	< 0.001	2.87	1.04	2.77	**0.006**
**Child displaying internalizing behavior at 3 years postpartum**								
No	3.91	1.03	3.79	< 0.001	3.02	1.03	2.94	**0.003**
**Rothbart temperament scale: effortful control**								
Medium/high	5.53	1.12	4.57	< 0.001	4.57	1.20	3.080	**< 0.001**
***Flood co-variants***								
**Impacted by the flood?**								
At least somewhat	1.26	1.06	1.19	0.23	–	–	–	–
**Flood preparedness**								
Unprepared	1.29	0.97	1.32	0.18	–	–	–	–
**Did you evacuate household?**								
Yes	0.98	3.09	0.46	0.64	–	–	–	–
**Did you or someone in household experience loss of income?**								
Yes	−0.46	1.13	−0.34	0.73	–	–	–	–
**Experienced threat or danger during flood?**								
Yes	0.69	1.87	0.37	0.71	–	–	–	–

*Partner relationship, history of mental health and mother’s education were not significant in the final model, hence were removed

### Multivariate analysis

The results of the multivariate linear regression model are shown in Table 3 Model B. No relationship was found between mother’s education, family income, mother’s ethnicity, marital status, delivery mode and child’s age. Maternal variables that contributed significantly to the model were: if mother’s age was 25 years or more, (B = 2.86, *p* = 0.04), and she had adequate social support (B = 2.50, *p =* 0.04). Child’s variables that contributed significantly included child’s sex as a girl (B = 1.57, *p* = 0.06) and if a child did not display internalizing (B = 3.02, *p* = 0.003) and externalizing (B = 2.87, *p* = 0.006) behavior problems, and if child had medium to high effortful control on Rothbart temperament scale (B = 4.57, *p =* < 0.001). The results indicated that on average, children of mothers aged > 25 years reported DESSA resilient score that was 2.86 points higher than the children of mothers aged < 25 years. Girls reported a resilient score that was 2.50 points higher than the boys. Furthermore, if mothers had adequate social support, children reported resilience score 2.50 points higher than the children whose mothers did not have adequate social support. Similarly, children reported 3.02, 2.87, 4.57 points higher resilient score if they did not display internalizing, externalizing behaviour problems, and had medium to high effortful control, respectively. Partner relationship, history of mental health, mother’s education were not significant in the final model, hence were removed from the multivariate analysis.

## Discussion

For this study, data from a community-based cohort were utilized. To our knowledge, this study is one of the first to have pre-disaster data to predict the influence of a natural disaster on child resilience within the context of pre- and post-disaster individual- and family-level characteristics. Lochman et al. used a similar study approach with a longitudinal cohort of low-income, primarily male, African-American 9- to 11-year old children who had been studied before and after a tornado in Alabama, US. However, this study did not have early life data (e.g., prenatal, postnatal), and the measure of resilience was aggression and internalizing problems [42]. Majority of children demonstrated resilient characteristics with 83% scoring in the normal range on the DESSA socio-emotional score. A higher DESSA score indicates greater social-emotional competence (resilience) and a lower score indicates lower social-emotional competence (non-resilience). In the current study, we did not use DESSA cut-off score but rather continuous scale was used in the model. We speculate that resilience (DESSA score) may vary among children belonging to different communities and families and under different type of adversity such as war, natural disaster, orphanage etc. This study indicated that children developed more resilience if the families were more impacted by the flood. We surmise that access of resources, disaster response, and recovery activities may have contributed to the greater maternal reported resilience in children impacted by the flood [43].

Eden Cohort (*N* = 1002) reported 10% non-resilient children whose language skills were delayed at age 3 years [44]. However, one study from the Avon Longitudinal Study of Parents and Children (*N* = 1009) reported 68% of children as non-resilient. These children did not develop behavioral and emotional problems at age 11 years despite exposed to postnatal maternal depression [45]. A possible explanation of this difference could be the variation in conceptualizing resilience, with language as a factor in the former study and behavioral-emotional data in the latter. For instance, a study from the same ALSPAC cohort (*N* = 7712) reported 13% of children as non-resilient. This study conceptualized resilient children as those who didn’t develop child peer problems at age 4 years despite exposed to intimate partner violence (IPV) [46].

The association between externalizing behaviour and non-resilience has not been assessed previously, likely because externalizing behaviour has been used as a proxy measure for resilience. However, given that resilient children demonstrate self-control and inhibition in the face of difficulty – characteristics that are lacking in those who exhibit externalizing behaviour – it is perhaps not surprising that non-resilience is 3-fold higher in those with externalizing behaviour. From an adaptive perspective, children may externalize in response to challenging situations. Not having the internal psychological resources linked to resilience, they display their frustrations in aggression, conduct disorder, or hyperactive behaviour [47, 48].

The study showed significant association between social support and child resilience. Previous studies have reported significant association between social support and child resilience. For instance, La Greca and colleagues examined children’s risk and resilience factors following a natural disaster and found social support as a protective factor against posttraumatic stress disorder in children exposed to a disaster [49]. Another Canadian study conducted in a community-based (N ~ 700) setting investigating early childhood risk and resilient factors reported adequate social support as a protective factor against risk of behavioral problems in children aged 6–8 years [50].

Our findings showed significant association between maternal history of mental health, child’s mental and physical health and resilience. Positive mental health is of paramount importance in managing stress, problem solving, and in times of facing adversity without disintegration [51]. The lack of direct relationship between child resilience and early prenatal and postnatal factors may suggest that these variables likely play an indirect role. A community-based mother-infant dyads study (*N* = 501) in Canada reported indirect effects of maternal adverse childhood experiences on children physical and emotional health at age 18 months [52]. Although, the study did not investigate child resilience, we surmise that early prenatal and postnatal factors played an intermediary role.

The current study has the following limitations. First, study measures including child resilience were mother-reported using standardized symptom measures versus observational and clinical assessment. Ideally, to strengthen the findings, child resilience would have been based on more objective measures and responses from multiple respondents including fathers, and teachers. Second, the study did not report mothers’ current mood which also plays an important role in child resilience [53]. Finally, while efforts were made to retain a population-based sample, there was low participation (~ 28%) in the current study compared to the study participants at intake. This attrition diminishes the external validity of this study and warrants caution when applying results to general populations, such as non-English speakers, people living with low income etc.

## Conclusions

The current study highlights the risk and protective factors that predict child resilience amidst early life adversity. These findings show that the children facing flood as natural disaster in early childhood period develop greater resilience to the adversity. Taken together, the present study supports the assertion that it is vital to improve child resilience in a community-based setting undergoing life adversities and a natural disaster e.g. interventions may be used to mitigate child externalizing behavioral problems. Furthermore, strategies need to be devised to foster social support, and reduce parental concerns regarding their child physical and mental health in conventionally low-risk families. Furthermore, a detailed analysis of child resilience remains an important study topic, as it will be of key interest to see how genetics and environmental factors play roles in improving child resilience amidst early life adversities.

## Data Availability

Data that supports the findings of this study are available from the PURLS dataset within the *Centre* for Health *Genomics* and Informatics (CHGI), Cumming School of Medicine, University of Calgary, but restrictions apply to the availability of supporting All Our Family data, which were used under license for the current study, and so are not publicly available. Data are however available from the authors upon reasonable request and with permission of All Our Families.
